# Age-related differences in humoral and cellular immune responses after primary immunisation: indications for stratified vaccination schedules

**DOI:** 10.1038/s41598-018-28111-8

**Published:** 2018-06-29

**Authors:** Angelika Wagner, Erika Garner-Spitzer, Joanna Jasinska, Herwig Kollaritsch, Karin Stiasny, Michael Kundi, Ursula Wiedermann

**Affiliations:** 1 Institute of Specific Prophylaxis and Tropical Medicine, Medical University of Vienna, Vienna, 1090 Austria; 2 Center of Virology, Medical University of Vienna, Vienna, 1090 Austria; 3 Institute of Environmental Health, Medical University of Vienna, Vienna, 1090 Austria

## Abstract

Immunosenescence is characterised by reduced B and T cell responses. Evidence shows that booster vaccinations are less effective in elderly people, but data on the efficacy of primary immunisation are sparse. We conducted a monocentric, open label, phase IV trial to compare immune responses to primary vaccinations using the inactivated, adjuvanted Japanese Encephalitis vaccine by 30 elderly people (mean 69, range 61–78 years) and 30 younger people (mean 24, range 18–30 years). Humoral and cellular immune responses were analysed in relation to age and cytomegalovirus (CMV) seropositivity. Vaccine-specific antibody titres were significantly lower in elderly participants and 47% of them were non- or low responders after the two doses of the vaccine neo-antigen. The reduced humoral immune responses in elderly people correlated with reduced cytokine production, such as interferon gamma (IFN-γ) *in vitro*, as well as higher frequencies of late-differentiated effector and effector memory T cells and T regulatory cells. These cellular changes and lower antibody titres were particularly prominent in CMV-seropositive elderly participants. If primary vaccination before the age of 60 is not possible, elderly patients may require different vaccination strategies to ensure sufficient long-lasting immunity, such as adapted or accelerated schedules and the use of different adjuvants.

## Introduction

Worldwide demographic changes are associated with an increasingly aging population. It is estimated that by 2050^1^ more than 20% of the global population will be over the age of 60 and this poses a growing challenge for public health systems to promote healthy aging societies.

Age-associated immune remodelling, together with other predisposing factors such as malnutrition, have showed that altered barrier function and chronic medical conditions lead to elevated susceptibility to infectious diseases^2^. This means that effective vaccines are an important tool for preventing infections in elderly people^3^. However, several studies that were primarily performed on booster responses to vaccines have reported that the immune responses were less effective in the elderly^4,5^. Lower antibody concentrations, with higher decline rates below seroprotective margins, resulted in an overall shortened period of protection^6,7^. These observations have been linked to the physiological impairment of the immune system, which is known as immunosenescence. This particularly affects the adaptive arm of the immune system, resulting in decreases in the B and T cell repertoire and the naive cell pool, as well as a restricted diversity increase in the memory and terminally differentiated T effector cells^8,9^. As a result of more rapid declines in antibodies with advancing age, health authorities in several countries, including Austria^10^, have recommended that elderly people receive boosters of a number of routine vaccines at shorter intervals than younger individuals.

In general, vaccine responsiveness and non-responsiveness is evaluated by measuring antibody titres. However, cellular responses can provide further information on vaccine efficacy^11^ and indicate protection as well as humoral responses. We previously evaluated vaccine responsiveness in different risk populations and demonstrated that non-responsiveness to selected vaccines did not show a uniform picture^12^. For example, genetically prone hepatitis B non-responders had strongly impaired cellular responses to booster vaccines other than hepatitis B and these did not correlate with humoral responses. In contrast, non-responders to the tick-borne encephalitis (TBE) booster vaccination only showed impaired responses to this particular vaccine, but these occurred at both humoral and cellular level^12^. We particularly noticed that the number of low and non-responders to booster vaccinations increased with age^12,13^, but very little information is available about the extent to which humoral and cellular responses are affected by primary vaccinations in the elderly population.

Since the ageing immune system may be confronted with previously un-encountered vaccine antigens, as newly developed vaccines become available or when people receive certain pre-travel vaccinations for the first time, we performed a study to determine age-associated differences in the humoral and cellular immune responses to a primary vaccination with a neo-antigen. For this purpose we selected a travel vaccine that is provided to protect against the Japanese encephalitis virus (JEV), as this flavivirus has restricted endemicity to Asia and the Western Pacific and our Austrian study subjects had not been previously been exposed to it. The vaccine antigen is adsorbed to aluminium hydroxid, an adjuvant that is well tolerated and therefore most widely used in human subunit and protein toxin vaccines in order to enhance humoral but to a lower extent cell-mediated immune responses (CD4+/T follicular helper cells)^14–16^.

This study presents data from our monocentric, open-label clinical trial that evaluated antibody production and T effector cell function, namely cytokine production, after two age groups, with mean ages of 24 and 69 years, received primary vaccinations against the JEV neo-antigen. The study aimed to provide further information on whether current vaccination strategies with neo-antigens were sufficient for the elderly. We also wanted to explore whether changes in either the schedule or vaccine doses/formulation might be necessary, in terms of a stratified vaccinology approach, to improve primary vaccine efficacy for elderly people.

## Results

### Demographic data

The mean age of the 30 elderly participants was 68.6 years (range 61–78 years) and it was 24.3 (range 18–30) in the 30 younger participants (Table 1). All the subjects were of Caucasian origin. All of the elderly subjects and 27 of the 30 younger participants had received TBE vaccinations in the past. Three of the 30 elderly participants had received a yellow fever (YF) vaccine more than 10 years ago, but none of the younger subjects had received this vaccination. We noted that 10% of the elderly and 16.7% of the young study subjects were current smokers and the body mass index (BMI) was higher in the elderly study participants, with a mean of 26.1, compared to 22.4 in the younger group. All 23 of the elderly participants with a chronic underlying disease took regular medication, but without immunosuppressive effects, whereas in the younger study group 14 out of the 30 participants had a chronic underlying disease and 12 required medication. However, they were still included as they complied with the inclusion and exclusion criteria of the study (see Supplementary Table S1).

**Table 1:** Patient’s characteristics.

	young	elderly
Participants	n = 30	n = 30
Gender (m/f)	15/15	15/15
Mean age	24,3 (18–30)	68,6 (61–78)
TBE immunisation (yes/no)	27/3	30/0
YF immunisation (yes/no)	0/30	3/27
Smoking (yes/no)	5/25	3/27
BMI (±SD)*	22,41 (±3,1)	26,06 (±3,3)

*BMI = kg/m^2^; SD = standard deviation.

### Lower JE vaccine specific neutralisation titres in the elderly

**Figure 1:**
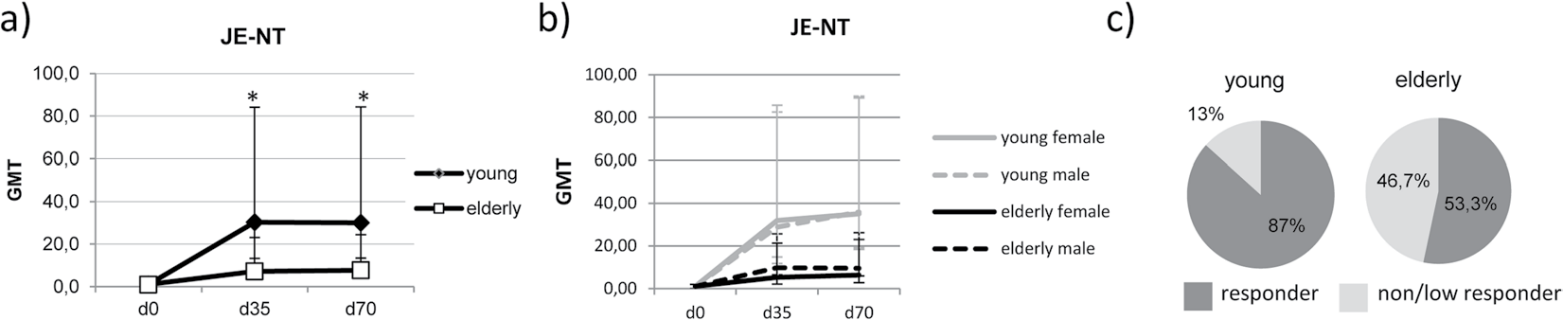
Vaccine-specific antibody titres. (**a**) Vaccine-specific JE virus (JEV-specific) antibody titres measured by neutralisation test (NT) in blood samples drawn on days 0, 35 and 70. Results are expressed as geometric mean titres (GMT) with 95% confidence intervals. (**b**) Vaccine-specific GMTs according to gender. (**c**) Proportions of vaccine responders (titre ≥ 1:20) and non/low responders (titre < 1:20) to JE vaccination. Statistical analysis by General Linear Model with log-transformed values. Individual comparisons by linear contrasts. *p < 0.05.

We assessed the antibody levels after the primary JE vaccination by measuring the neutralising antibody titres (NT) in order to avoid cross-reactivity between the JEV, TBEV or YF specific immunoglobulin G (IgG) antibodies in the enzyme-linked immunosorbent assay (ELISA). Pre-vaccination JEV titres were below the detection limits in all study participants, confirming that none of them had previously been exposed to JEV (Fig. 1a). After the primary JE vaccination, significantly lower geometric mean titres (GMT) were detected in the elderly group on days 35 and 70 than in the young group. However, no significant differences in antibody titre levels were detected between days 35 and day 70 within each age group and we did not detect significant gender-related differences in antibody levels (Fig. 1b). The percentage of non and low responders exhibiting antibody titres of <20 on days 35 and 70, were significantly higher in the elderly group (46.7%) than the young group (13%) (Fig. 1c).

### Altered JE vaccine -specific cytokine responses in the elderly

**Figure 2:**
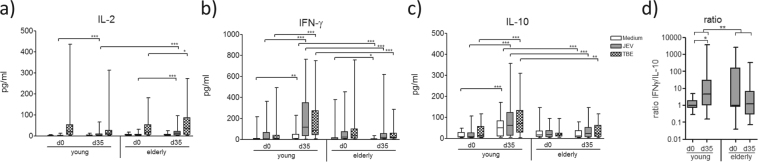
Vaccine-specific cytokine production. (**a**) IL-2, (**b**) IFN-γ and (**c**) IL-10 production of PBMC after 48 hour stimulation *in vitro* with JE virus antigen and TBE virus antigen (TBEV), respectively, or incubation with just medium. Cytokine levels were measured in supernatants. (**d**) JE-specific IFN-γ/IL-10 ratio after subtraction of medium values. Statistical analysis by General Linear Model with log-transformed values. Individual comparisons by linear contrasts. *p < 0.05; **p < 0.01; ***p < 0.001.

Cytokine production in response to medium, JEV and TBEV antigen stimulation was detected in supernatants of restimulated peripheral blood mononuclear cells (PBMC) before and after the primary JE vaccination, on days 0 and 35. Stimulation with the JEV antigen significantly increased interleukin 2 (IL-2) levels from day 0 to day 35 in both study groups (Fig. 2a). No significant difference in IFN-γ levels before and after vaccination were detected in the elderly group in response to JEV antigen stimulation, but a significant increase in IFN-γ levels between days 0 and 35 was present in the young group (Fig. 2b). IL-10 levels did not significantly differ before and after primary JE vaccinations in the elderly, but they increased in the young group from days 0 to 35 (Fig. 2c). The JE-specific IFN-γ/IL-10 ratio showed a significant difference between groups over time with a significant shift towards Th1 in the young versus a trend towards Th2 in the elderly (Fig. 2d).

### Dissociation of TBEV specific humoral and cellular responses after primary JE vaccination in the elderly

All study participants, except three in the young age group, have received TBE vaccinations previously. Based on the known cross-reactivity between JEV and TBEV-specific IgG^17^, we tested whether cross-reactivity also existed at the T cell level between the two antigens. We first evaluated if TBEV-specific neutralising antibodies were influenced by the JE vaccination. When we compared TBEV-specific GMT before and after the primary JE vaccination we did not observe significant changes in neutralisation titre levels (Table 2). The TBEV-specific titres were significantly lower in the elderly group, compared to the young group, at all evaluated time points (days 0, 35 and 70), even though the mean interval to the last booster was shorter (2.0 years) in the elderly group than young group (3.8 years).

**Table 2:** TBE-specific GMT and 95% confidence intervals.

	Day 0 GMT (95% CI)	Day 35 GMT (95% CI)	Day 70 GMT (95% CI)
young	58,19 (34,27–82,11)	55,56 (13,51–97,61)	50,00 (18,07–81,89)
elderly	31,73 (0–100,56)**	30,13 (0–83,17)**	26,48 (0–84,88)**

Statistical analysis by General Linear Model with log-transformed values. Comparisons between young and elderly by linear contrasts. **p < 0.01.

With regard to the cytokine levels in TBEV-antigen stimulated PBMC cultures before and after the primary JE vaccination, IL-2 levels increased in both study groups between days 0 and 35 (Fig. 2a), reaching significantly higher amounts on day 35 in the elderly group. IFN-γ and IL-10 remained unchanged in the elderly group and significantly increased from day 0 to day 35 in the young group (Fig. 2b,c).

### Characterisation of cellular compartments

#### Redistribution of naive towards memory B cell subsets in the elderly

**Figure 3:**
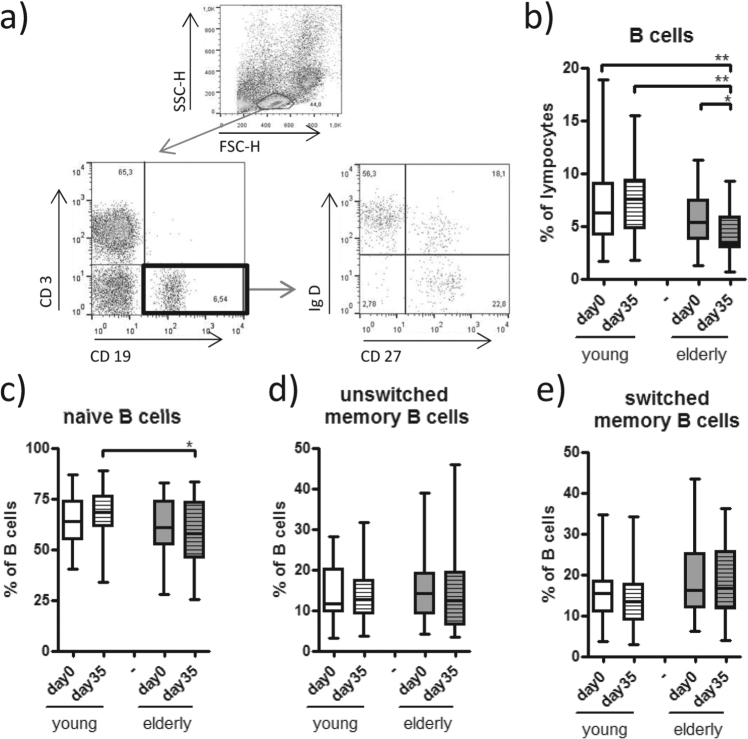
B cell subsets. (**a**) The percentage of B cells (CD3− CD19+) was determined after staining of PBMC, derived on days 0 and 35, with CD3, CD19, CD27 and IgD and gating on the live lymphocyte population in a SSC/FSC blot. (**b**) Further evaluation of naive (CD27− IgD+), (**c**) unswitched (CD27+IgD+) (**d**) and switched memory B (CD27+IgD−) cells were performed according to the expression of CD27 and IgD on gated B cells. Statistical analysis by General Linear Model with arcsine-transformed percentages. Individual comparisons by linear contrasts. *p < 0.05; **p < 0.01.

Immunosenescence includes alterations in the B cell subset, with a decline of naive B cells leading to an expanded pool of antigen experienced B cells^8,18^. We detected a significantly lower percentage of B cells (CD19+ CD3−) in the lymphocyte subset in the elderly group before and after primary vaccination compared to the young vaccinees (Fig. 3b). With regard to different B cell subsets, the percentages of naive B cells were markedly reduced on day 35 (Fig. 3c), whereas the switched memory B cells tended to be increased (*p* = 0.1) in the elderly group compared to the young group (Fig. 3e). After vaccination we detected a significant decrease in B cells in the elderly whereas B cells increased non-significantly in the young (Fig. 3b).

#### Shift from naive to memory T cell subsets in the elderly

**Figure 4:**
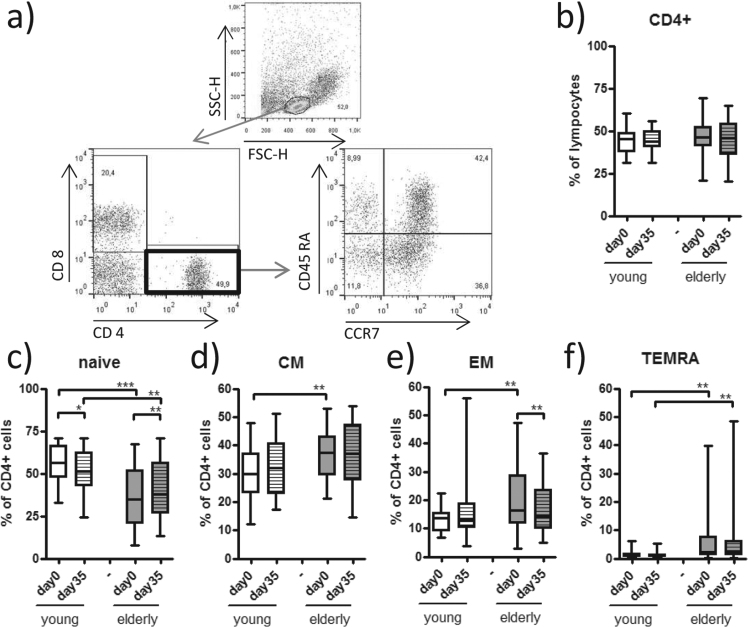
Naive and memory CD4+ cell subsets. (**a**) CD4+ T cells were identified as CD4+CD8− lymphocytes within the live lymphocyte population in a SSC/FSC blot of surface stained PBMC obtained on days 0 and 35. (**b**) Concomitant staining with CD45RA and CCR7 allowed differentiation of naive CD4+ T cells (CD45RA+CCR7+), (**c**) central memory (CM) CD4+ T cells (CD45RA− CCR7+), (**d**) effector memory (EM) CD4+ T cells (CD45RA− CCR7−) and (**e**) CD4+ T effector memory cells that re-express CD45RA (TEMRA) (CD45RA+ CCR7−) within the CD4+ T cells. Statistical analysis by General Linear Model with log-transformed values. Individual comparisons by linear contrasts. *p < 0.05; **p < 0.01; ***p < 0.001.

It has been shown that, in addition to the age-related alterations in the B cell compartment, the distribution of both the CD4+ T helper cell and the CD8+ cytotoxic T cell subpopulations undergo changes with increasing age^19^. In our study, CD4+ T cells had a comparable distribution in the lymphocyte compartment in both age groups (Fig. 4b). However, the subpopulation analysis revealed that naive CD4+ T cells were significantly lower in the elderly group than young group (Fig. 4c). Regarding the CD4+ memory subsets, the elderly group exhibited higher frequencies of central and effector memory CD4+ cells and these were most prominent on day 0 (Fig. 4d,e). The CD4+ TEMRA population was significantly higher in the elderly before and after the primary vaccination (Fig. 4f).

We found lower frequencies of CD8+ T cells in the lymphocyte compartments of the elderly group than young group before and after the primary vaccination (see Supplementary Fig. S2a). Furthermore, we measured significantly less naive CD8+ T cells versus a significantly increased CD8+ TEMRA population in the elderly group at both time points (see Supplementary Fig. S2b,e). Central and effector memory CD8+ cells were slightly increased, although not significantly, in the elderly group (see Supplementary Fig. S2c,d). In the elderly participants, a higher percentage of CD4+ and CD8+ T cells had already lost CD27 and CD28 surface co-stimulatory molecules, resulting in lower frequencies of early-differentiated effector cells, along with higher percentages of late-differentiated effector CD4+ and CD8+ T cells on day 0 and 35 (see Supplementary Fig. S3a,c,d,f). Intermediately-differentiated effector T cells were increased among the CD4+ cells in the elderly group, whereas this differentiation stage in the CD8+ subset was at comparable levels between both groups at both time points (see Supplementary Fig. S3b,e). Upon vaccination we noted a decrease in naive CD4+ T cells in the young (Fig. 4c). In contrast, in the elderly we detected an increase in naive CD4+ T cells (Fig. 4c) and CD8+ TEMRA cells (see Supplementary Fig. 2e), as well as a decrease of CD4+ EM T cells (Fig. 4e) after vaccination.

#### Regulatory T and B cells

**Figure 5:**
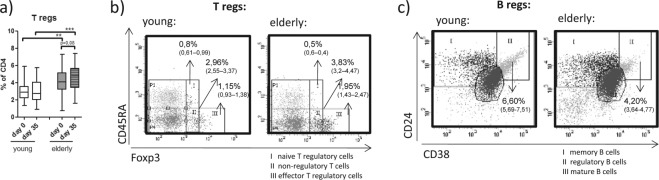
T and B cells with regulatory activity. (**a**) Regulatory T cells (CD25+ Foxp3+) were analysed by gating on the CD4+ live lymphocyte population. Additional staining with CD45RA allowed the sub-differentiation of regulatory T cells into (**bI**) naive (CD45RA+Foxp3^low^), (**bII**) non-regulatory (CD45RA- Foxp3^low^) and (**bIII**) effector (CD45RA- Foxp3^high^) CD3+CD4 + T cells within the live lymphocyte population. (**c**) Immature transitional B cells with potential regulatory activity were characterized as CD24^high^CD38^high^ gated B cells (CD3-CD19+) within the live lymphocyte population (**cII**). Sub-differentiation of T and B cells was performed in blood samples obtained on day 70. Statistical analysis by General Linear Model with arcsine-transformed percentages. Individual comparisons by linear contrasts. **p < 0.01; ***p < 0.001.

Since regulatory T and B cells have been shown to modulate immune responses to unrelated antigens via cytokine production or cell-cell contact^20^, we characterised these two cell subsets in our study population. The percentages of regulatory T cells (CD4^+^CD25^+^Foxp3^+^) were significantly higher in the elderly than in the young group, with a trend (*p* = 0,08) towards higher regulatory T cells after vaccination in the elderly (d 35) (Fig. 5a). The regulatory T cell population can be further divided into subsets according to their grade of differentiation and suppressive activity^21^. Our study shows that moderately suppressive naive regulatory T cells significantly decreased with age (*p* < 0.05), whereas the percentages of highly suppressive effector regulatory T cells markedly increased (*p* < 0.01; Fig. 5b). Interestingly, non-regulatory T cells tended to increase in the elderly (*p* = 0.05; Fig. 5b), while immature transitional B cells - a potentially B regulatory cell population able to mediate suppression via IL-10 production or cell-cell contact^22^- were significantly reduced in the elderly group compared to the young group (*p* < 0.001) (Fig. 5c).

### CMV-seropositivity was associated with altered humoral and cellular immune responses in the elderly

**Figure 6:**
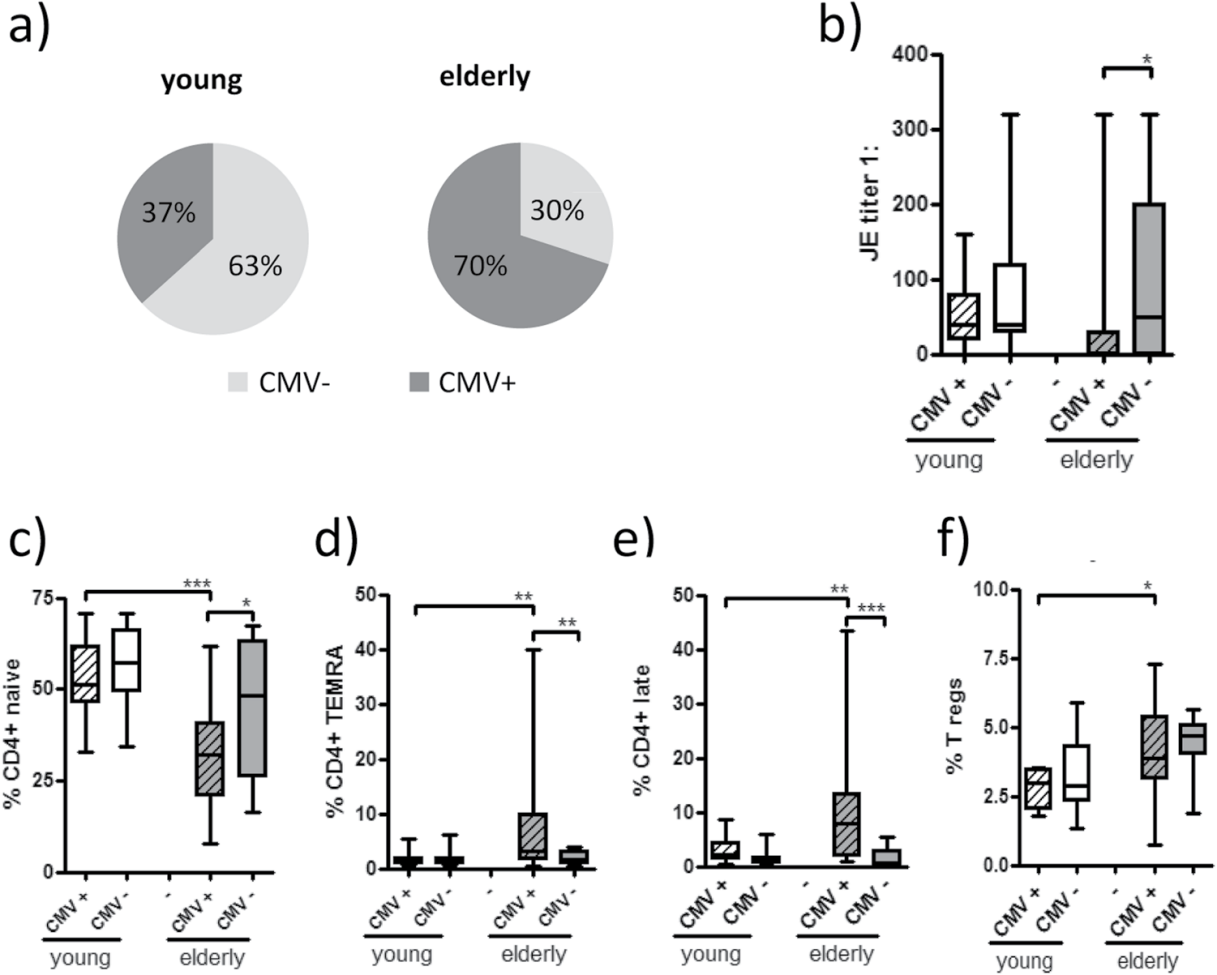
Antibody titre and distribution of different T cell subsets in CMV seropositive and seronegative participants. (**a**) CMV-specific antibody titres were measured by ELISA in sera obtained on day 0. Results of RU >16 were considered positive and individuals categorised as seropositive. (**b**) JEV-specific neutralising antibody titres (day 35), (**c**) CD4+ naive, (**d**) TEMRA, (**e**) late-differentiated effector cells and (**f**) regulatory T cells were analysed according to CMV serology results (all cell subsets were measured in samples obtained on day 0, for gating strategy see Figs 3 and 4). Statistical analysis by General Linear Model with arcsine-transformed percentages. Individual comparisons by linear contrasts. *p < 0.05; **p < 0.01; ***p < 0.001.

Since infection with the CMV has been shown to influence the age-related modification of adaptive immunity^23^, CMV serology was performed on all study participants and this showed that 70% of the elderly group and 37% of the young group were CMV-seropositive (Fig. 6a). When we evaluated humoral and cellular immune responses according to CMV sero-status, we detected significantly lower JEV-NT titres in the CMV-seropositive elderly participants (Fig. 6b). Interestingly, IL-2 levels were particularly higher in the elderly participants who were seronegative for CMV (mean 38.0 pg/ml) than those who were seropositive (mean 12,4 pg/ml; *p* < 0.01; data not shown). When we analysed the T cell subset distributions in relation to CMV sero-status, CMV-seropositive elderly participants displayed significantly lower percentages of naive CD4+ T cells, whereas CD4+ TEMRA and late-differentiated T cells were markedly increased compared to CMV-seronegative elderly subjects (Fig. 6c–e). While the percentage of regulatory T cells was significantly higher in the elderly than the young (Fig. 5a), no difference was seen between CMV-seropositive and negative elderly subjects (Fig. 6f).

With regard to the CD8+ T cells, CMV-seropositive elderly subjects exhibited significantly lower percentages of naive CD8+ T cells (see Supplementary Fig. S2f), but markedly higher CD8+ TEMRA and late-differentiated CD8+ cells compared to the CMV-seronegative elderly (see Supplementary Fig. S2g,h). In the young CMV-seropositive versus CMV-seronegative vaccinees, no differences – except for CD8+ late differentiated T cells – was seen in the T cell subpopulations.

### Correlation between JE antibody levels and cellular responses

There was a positive correlation, though without significance, between vaccine-specific antibody levels and vaccine-specific IFN-γ levels in both age groups (see Supplementary Fig. S4a). Additionally, we detected an inverse, yet non-significant, correlation between an increase in T regulatory cells post vaccination and vaccine-specific antibody titres after vaccination in the elderly as well as the young vaccines (see Supplementary Fig. S4b).

## Discussion

This study investigated whether humoral and cellular immune responses following primary vaccination were affected by age. It also determined what impact the findings could have on current vaccination recommendations and schedules to guarantee sufficient protection against vaccine preventable diseases in elderly and geriatric age groups. The JE vaccine we tested served as a model vaccine with a neo-antigen to which none of the recruited elderly or young study participants had previously established immunity.

Neutralising JEV-specific antibodies has been proposed as a correlate of protection based on clinical and murine data^24,25^, showing that protection increases with respective NT titres^26^. In our study, we detected that the elderly group had a reduced capacity to produce neutralising antibodies following primary vaccination against the JEV. Accordingly, the proportion of non and low responders was significantly higher in this group than in the young group. These results seem to be in contrast to the licensing studies, where age-related differences in seroconversion and GMT were not found in subjects above and below 50 years after primary vaccination^27^. One reason for this could be the different assays used in the licensure study, which had a lower-cut off level (<1:10) than our study (<1:20) for identifying non/low responders. Another reason could be the more defined age stratification between the participants in our study, i.e. including young participants at a mean age of 24 (range 18–30) years and elderly people at a mean age of 69 (range from 61–78) years. Because of these significant age differences, the age-related differences in vaccine responsiveness were much more obvious in our study than in a population that included participants in their 40 s and 50 s, during which the primary vaccine responses may only just be starting to decline, as shown for hepatitis B immunisation^28,29^. Another aspect worth considering in relation to reduced vaccines responses in the elderly is seropositivity to the cytomegalovirus (CMV), as CMV has been associated with age-dependent rearrangement of T cell subpopulations and an increase in antigen-experienced lymphocytes^30^. In our study population, the percentage of seropositive individuals clearly increased with age and this corresponded with worldwide seroprevalence data^31^. Indeed, when we tested whether the altered vaccine responses were associated with CMV seropositivity, we found that CMV-seropositive elderly subjects had significantly lower antibody titres after they received the primary JE vaccination than the elderly CMV-seronegative vaccinees. So far the existing data on other vaccines given to elderly people, such as influenza, have been contradictory with regard to the effects of CMV infections on humoral responses^32–36^. However, differences in influenza-antibody titres might be due to possible previous exposure to some influenza vaccine strains (or drift variants) in the elderly and therefore may reflect a mixture of recall and primary responses. Nevertheless, our data support the theory that the CMV infection contributed to the dramatic age-related difference in antibody titres after the primary vaccination. This indicates that long-term protection could also be affected and might even serve as a prediction marker of vaccine responsiveness in the elderly.

It has been established that the pool of naive B cells, and the diversity of B cell receptors, decrease with age^9^ and that the ability to form germinal centres is also reduced^8^. In our study, we confirm a lower percentage of total and naive B cells in the elderly group compared to the young group. Therefore, it might be assumed that the lower antibody titres in this group were due to the lower number of naive B cells that were able to encounter their corresponding antigens. Moreover, the highest percentage of switched B memory cells, an antigen-experienced cell population no longer involved in primary responses, were also found in the elderly group.

Cross-reactive antibodies to different flaviviruses, such as the West Nile virus and dengue virus type 2, have been described in individuals vaccinated for YF, TBEV and JEV^37,38^. With regard to JEV and TBEV, we did not detect cross-neutralising antibodies against JEV in any of the TBE-primed subjects before JE vaccination in our study population. We had previously found higher JEV-specific titres in TBE-vaccinated participants, but only transiently after the first JE vaccine dose^39^. Our current observation that primary vaccination against the JEV did not change TBEV-specific titre levels supports the notion that there is no cross-neutralisation at the humoral level between these two flaviviruses.

In addition to the JEV-specific humoral responses after the primary vaccination, the cellular responses were also altered in the elderly group. Surprisingly, IL-2 production increased in the young and the elderly, with the highest levels in the elderly CMV-seronegative subset. As well as its known lymphoproliferative function, IL-2 has been shown to have the ability to block T follicular helper cell differentiation^40^. This means that our findings would fit with lower antibody titres in the elderly. Moreover, this cytokine is important for T regulatory cell induction in the periphery, as well as their survival^41^, which was significantly increased in the elderly participants. In contrast we only found increased IFN-γ levels between day 0 and day 35 in the young, but the lack of IFN-γ production in the elderly after vaccination reflected the significantly reduced population of naive CD4+ lymphocytes in this age group. The importance of IFN-γ in controlling JEV-induced encephalitis has previously been reported in a model with IFN-γ KO mice^42^. In humans, the ability of PBMC to produce higher IFN-γ levels correlated with better clinical outcomes after the JEV infection^43^. This raises the question about whether – in correlation with the lower NT antibody titres - lower vaccine induced IFN-γ production may indicate lower vaccine-induced protection in the elderly. Along these lines, the JE-specific IFN-γ/IL-10 ratio tended to decrease in the elderly while it significantly increased in the young. These results further indicate that the immune responses in the elderly could be improved by the use of Th1 promoting vaccine formulations, as also discussed by others^44,45^.

Interestingly, and in contrast to the humoral responses, TBEV-specific cytokine responses increased concurrently with JEV-specific cytokine production after primary JE-vaccination as seen for IL-2 in the elderly and for IFN-γ as well as IL-10 in the young. Since the majority of the study participants have had several TBE vaccinations in the past (due to the high TBEV endemicity), the parallel rise in cytokines might be due to antigenic cross-reactivity at the cellular level between JEV and TBEV antigens as previously described for other flaviviruses^46,47^. Consequently, the increased IL-2 levels, detected only in the elderly, might have resulted from more frequent TBE vaccinations or exposure in the elderly compared to the young.

Vaccine-induced immune protection and viral defence mechanisms against JEV depend on effector CD4+ and CD8+ cell subsets^46^. Although the CD4+ T cells were rather sustained at the time of the vaccination in our study cohort, the CD8+ T cells had already declined in the elderly participants, confirming previous observations^19^. Importantly, we noted a reduction of naive T cells, both CD4+ and CD8+, versus an increase of the end-stage differentiated TEMRA in the elderly. Similarly, the early-differentiated T cells, which also included the naive T cells, were diminished, but the late-differentiated T cells, which encompassed the TEMRA lymphocytes, predominated. The shift from naive to late or end-stage differentiated T cell subpopulations became even more evident in the CMV-seropositive elderly subjects. Therefore, our results are in agreement with the concept of chronic CMV infection being a major driver of immunosenescence^32,48^. They also complete the picture that elderly people are very probably less able to mount an adaptive and protective response against a neo-antigen and this ability decreases even more if they are CMV seropositive.

Apart from the redistributed lymphocyte subsets in the elderly, T and B regulatory cells, which are known for their active immunosuppressive capacity, might be responsible for altered vaccine responses^49^. In this respect, we previously noted that T regulatory cells expanded in hepatitis B as well as in TBE non-responders after booster vaccinations^12,50^ while a decrease in T regulatory cells was found in those mounting a sufficient humoral and cellular vaccine response^12,51^. In line with our previous studies we here demonstrate that the low vaccine response in the elderly is associated with increased regulatory T cells. Similarly, an inverse relationship between regulatory T cells and low vaccine responsiveness was also shown in certain risk groups such as cancer patients and HIV+^51,52^. Generally, T regulatory cells can mediate their regulatory potential via cell-cell contact or via cytokines such as IL-10 that can suppress cell activation and co-stimulation^20^. In accordance, the trend towards Th2/Treg responses as indicated by the reduced INF-γ/IL-10 ratio in the elderly was accompanied by increased levels of T regulatory cells.

T regulatory cells can be further classified via CD45RA into subsets, according to their suppressive potential, whereby effector T regulatory cells exhibit the highest suppressive capacity^53^. It was notable in our study that this subset was significantly increased in the elderly subjects after vaccination, making them the possible culprits for lower vaccine responses. However, the expansion of these cells was independent of CMV seropositivity, in contrast to another study that showed that the increase in regulatory T cells in the elderly was further enhanced by the CMV infection^54^.

We previously detected higher levels of immature transitional B cells, a potentially regulatory B cell subset able to suppress immune responses to unrelated antigens^55^, in vaccinated non-responders with a genetic predisposition to hepatitis B, but not in those without a genetic predisposition (TBE)^12^. However, in line with a study by Duggal *et al*.^22^, the elderly subjects who were vaccinated showed lower levels of immature transitional/B regulatory cells, thus indicating that this B cell subset is not associated with impaired vaccine responses in elderly individuals.

While the results expand previous findings in elderly vaccinees receiving a neo-antigen, there are limitations due to the exploratory nature of some of the analyses (impact of CMV and lymphocyte subsets) and the limited sample size. The study was powered to analyse 5 different endpoints at the 5% level of statistical significance. A comparison of elderly and young for all endpoints reported here, maintaining the same power and keeping the statistical error (type 1) at a constant level of 5%, would have required sample sizes between 250 and 750 per group depending on the effect size. It is therefore possible that some additional differences exist that were not detected due to insufficient power. There is, on the other hand, the risk that the probability of error is uncontrollably increasing due to multiple endpoint testing. These limitations, however, were accepted in favour of the feasibility of the clinical trial.

Taken together, our study findings demonstrate that decreased vaccine-specific humoral and cellular responses following primary vaccination with a neo-antigen were associated with reduced naive B and T lymphocytes and increased T regulatory cells in elderly subjects. A shift from naive to expanded antigen-experienced effector and memory T cell pools was particularly prominent in the elderly participants infected with CMV. The association between reduced vaccine responsiveness and redistributed B and T cell subsets in subjects with CMV infection suggests that CMV sero-status could serve as one possible biomarker for non or low responsiveness, particularly with regard to primary vaccination of the elderly.

To improve vaccine responses to neo-antigens in elderly and geriatric people, we need to adopt a number of strategies, including stratified vaccination schedules that adapt the existing antigen doses, route of application (i.e. intradermal), and/or accelerated immunisation schedules. Future vaccine development should target the use of more Th1-promoting adjuvants to enhance humoral and cellular vaccine-specific responses as well as long-lasting memory responses. The question of booster responsiveness after primary vaccination in the elderly is the subject of a continuing study.

## Methods

### Study population and study design

Having received written, informed consent we included in total 30 young volunteers aged 18 to 30 years and 30 elderly volunteers aged 61–78 years in our study (Table 1). We started recruiting in November 2011 until September 2012, with the last follow-up visit in November 2012 and all 60 participants completed the trial. The trial was closed as planned after the last study visit. The subjects were matched for gender with no prior history of the Japanese Encephalitis (JE) vaccination and/or infection with any of the flaviviruses. A vaccination history of TBE immunisation was acceptable if the last booster has been received at least 30 days before study inclusion as well as a YF vaccination at least 10 years before. According to our inclusion and exclusion criteria, we ensured that participants had no acute illness or chronic medical condition or treatment known to interfere with immune responses at the time of study inclusion and during the study period. This did not exclude well-controlled medical conditions, such as mild allergies, arterial hypertension, hypercholesteremia or diabetes type 2 (see Supplementary Table S1).

Participants received a primary course of a licensed inactivated Vero-cell based JE vaccine (IXIARO^®^, Valneva, Vienna, Austria) into the deltoid muscle and this contained 6 µg of the inactivated JE virus strain SA_14_-14-2 adsorbed to aluminium hydroxide. Two doses of the vaccine were administered four weeks apart, according to the standard immunisation protocol (see Supplementary Fig. S1). No harms in relation to the study medication were detected throughout the study period or reported thereafter. The study was performed as a mono-centric, open-label, phase IV study at the outpatient clinic of the Institute of Specific Prophylaxis and Tropical Medicine at the Medical University of Vienna. The trial was approved by the Ethics Committee of the Medical University of Vienna and the Austrian Agency for Health and Food Safety and the study was conducted according to the Declaration of Helsinki/International Conference on Harmonisation Guideline for Good Clinical Practice. The clinical trial was registered at ClinicalTrials.gov on 19^th^ of July 2011 (NCT01398540).

### Blood sample preparation

Blood samples were taken before the first vaccination (day 0) and then one week (day 35) and six weeks (day 70) after the second dose (see Supplementary Fig. 1). Serum and plasma samples were obtained after centrifugation (700 × g, 10 minutes) at all three time points and stored at −20 °C until analysis. PBMC were isolated from heparinised blood samples on day 0 and 35 after centrifugation (1300 rpm, 20 minutes) with Ficoll™, a density gradient-based lymphocyte separation medium (LSM 1077, PAA, Pasching, Austria). PBMC were either surface stained for flow cytometry analysis of different lymphocyte subsets or stored in liquid nitrogen until antigen-specific restimulation or further flow cytometry analysis.

### Antibody measurements

Neutralisation tests (NT) to determine JEV-specific neutralising antibodies were carried out in Vero cells (ECACC 88020401) using the Beijing JEV strain. Two-fold serial dilutions of heat-inactivated serum samples (obtained at days 0, 35 and 70) were incubated with 60–120 TCID50 virus for one hour at 37 °C. Cells were then added and incubation was continued for four days. The presence of any virus in the supernatant was assessed by the occurrence of cytopathic effects. JEV-NT titres of ≥20 were considered positive and participants exhibiting JEV-NT titres of <20 were considered as non or low responders.

TBEV-specific neutralising antibodies were measured by NT, as described previously^56^. Briefly, two-fold serial dilutions of heat-inactivated serum samples were incubated with the Neudoerfl TBEV strain (25 pfu) for one hour at 37 °C before baby hamster kidney cells (ATCC BHK-21) were added and incubation was continued for three days. The presence of any virus in the supernatant was assessed by ELISA. The virus neutralisation titre was defined as the reciprocal of the serum sample dilution that showed a 90% reduction in the absorbance readout in the assay when compared to the control without any antibodies. TBE-NT titre ≥10 were considered positive.

CMV-specific IgG antibodies were measured in serum samples obtained before the first vaccination by ELISA, according to the manufacturer´s instructions (Euroimmun Medizinische Labordiagnostika AG, Lübeck, Germany) and the results are expressed as relative units (RU), with measurements of <16 were being defined as negative.

Both the NT and CMV serology were performed at the Center of Virology of the Medical University of Vienna.

### Antigen-specific restimulation of PBMC for cytokine production *in vitro*

PBMC were thawed and re-suspended in RPMI-1640 medium supplemented with 10% human AB serum (Biochrom, Merck, Germany), 2mM L-glutamine (PAA), 50 µM 2-mercaptoethanol (Sigma Aldrich, St. Louis, MO, USA) and 0.1 mg/ml gentamycin (Sigma Aldrich). For re-stimulation, PBMC (8 × 10^5^/well) were incubated in a 96-well plate with the JEV antigen (12.5 µg/ml), TBE antigen (2.4 µg/ml), superantigen staphylococcal enterotoxin B (SEB; 1 µg/ml) or just medium in a final volume of 200 µl. The JEV antigen was kindly provided by Dr Klade, formerly of Intercell and now Valneva. The TBE antigen strain Neudörfl was provided by the company that was Baxter Innovation, Vienna, Austria and is now Pfizer. Plates were incubated at 37 °C and 5% of carbon dioxide for 48 hours. Thereafter, supernatants were harvested and stored at −20 °C until they were tested during the following days.

The quantification of IL-2, IFN-γ and IL-10 levels were performed by Luminex technology, according to the protocols, using the Fluorokine MAP Human Base Kit A (R&D Systems, Minneapolis, MN, USA) and a Luminex reader (AtheNA Multi-Lyte®, Zeus Scientific, Raritan, NJ, USA).

### Flow cytometry analysis

Isolated PBMC (5 × 10^5^) were first blocked with 20% of human AB serum in PBS containing 0.5% BSA and 0.5% sodium azide, subsequently referred to as FACS buffer, for 20 minutes. In order to distinguish between different B-cell and T-cell subsets, the following monoclonal antibodies were added for surface staining: anti-CD3-Pe-Cy5 (BD Pharmingen), anti-CD4 FITC (BD), anti-CD4-PE-Cy5 (BD), anti-CD8-APC (BD), anti-CD19-FITC (BD), anti-CD24-PE (BioLegend, San Diego, CA, USA), anti-CD27 PE (BD), anti-CD28-PE-Cy5 (BD), anti-CD38-PE-Cy5 (BioLegend), anti-CD45RA-PE (BD) and anti-CCR7-FITC (R&D Systems). For IgD staining, anti-human biotinylated IgD was used before Streptavidin-APC (BD). Regulatory T cells were first stained with anti-CD4 FITC (BD), anti-CD25-PR (BD), anti-CD45RA-BV510 (BD) in brilliant stain buffer (BD Biosciences, East Rutherford, NJ, USA), washed and incubated in fixation/permeabilisation buffer (eBioscience, San Diego, CA USA) before anti-Foxp3-APC staining (eBioscience) in permeabilisation buffer (eBioscience). After 30 minutes of staining, the cells were washed in FACS buffer before being analyzed on a FACSCalibur™ flow cytometer (BD Biosciences). Data were further evaluated using FlowJO software version vX.0.6 (Tree Star, USA). The cytometric analysis of the B and T regulatory cells from day 70 were performed with thawed PBMC on a FACS Canto™ II and the data were analyzed using FACS Diva software 8.0 (BD Biosciences). The gating strategy was performed as indicated in the figure legends.

### Statistical analysis

As primary endpoint the antibody titre at day 70 was defined, secondary endpoints included increase of JE-specific cytokine levels after vaccination. All other endpoints were exploratory. Based on prior investigations^57^ the standard deviation of log_e_ titres was assumed to be 0.5. With respect to previous study results^4,5^, a difference of 1:3 in titres of the elderly compared to the young study participants was deemed relevant. Based on these data the standardized effect size is 1. Due to the multiple endpoints sample size calculation was performed on the basis of a corrected alpha error of 0.01. To detect the specified effect with a power of 80% 26 subject need to be included per group. With an estimated dropout rate of about 10%, 30 subjects per group should be recruited.

All study participants enrolled (60 in total), received the intended study medication, completed the study and their results entered statistical analysis. Antibody titres are expressed as GMT with 95% confidence intervals (95% CI). Results for cytokines and lymphocyte subpopulations are shown as box-plots, with medians, interquartile and non-outlier ranges. However, for the statistical evaluation the cytokine concentrations were log-transformed and the percentages of the lymphocyte subpopulations were arcsine-transformed. The statistical analyses were carried out using the General Linear Model and the within subject factor was the time points of the blood withdrawals, the between-subject factors were the age groups, and CMV positivity in some of the analyses. These analyses were carried out on a multivariate basis for lymphocyte subpopulations, and if any variables showed significance in the multivariate analysis then univariate p values were computed for each of those variables. For contrasts Bonferroni correction was applied. Residuals were tested for deviation from normality by Kolmogorov-Smirnov tests with Lilliefors adjusted p values. Homogeneity of variances was tested by Levine’s tests and p values below 0.05 were considered significant. No adjustments for multiple endpoints were applied.

### Data availability

The authors are pleased to respond to reasonable requests for datasets analysed during the current study.

## Electronic supplementary material

Supplementary information
