# Supplementary material for: Age-related differences in humoral and cellular immune responses after primary immunisation: indications for stratified vaccination schedules
Source: Sci Rep. 2018 Jun 29;8:9825. doi: 10.1038/s41598-018-28111-8 (PMC6026142; doi:10.1038/s41598-018-28111-8)
Supplement: Supplementary file 1 — Supplementary information [file 41598_2018_28111_MOESM1_ESM.pdf]

Age-related differences in humoral and cellular immune responses after primary immunisation: indications for stratified vaccination schedules

Angelika Wagner, Erika Garner-Spitzer, Joanna Jasinska, Herwig Kollaritsch, Karin Stiasny, Michael Kundi, Ursula Wiedermann

Supplementary Table 1:
List of inclusion and exclusion criteria

Inclusion Criteria:

- Adults (18-40 years and ≥60 years with no upper age limit)
- Both sexes
- Individuals in good health conditions including patients with well-controlled mild allergies, arterial hypertension, hypercholesteremia or diabetes type 2
- Willingness to sign the written informed consent form

Exclusion Criteria:

- Age below 18 years, age between 41 and 59 years
- Pregnancy or breastfeeding
- Prior Japanese Encephalitis, TBE, dengue, yellow fever or West Nile/neuroinvasive disease virus infection
- Yellow fever vaccination within the last 10 years before the first dose of IXIARO® and during the study period (visit 1 until visit 4)
- TBE vaccination within the last 30 days before the first dose of IXIARO® and during the study period (visit 1 until visit 4)
- Vaccination against dengue or West Nile Virus
- Current acute illness (body temperature above 37,9°C) or exacerbation of underlying diseases at the day of IXIARO® vaccination (visit 1 and 3).
- Planned surgery within 2 weeks prior or post study medication i.e. IXIARO® vaccination
- Immunotherapy within 2 weeks prior or post study medication i.e. IXIARO® vaccination
- Plasma donors
- Blood transfusion or immunoglobulin within 3 months before study inclusion
- Participation at another clinical study with application of any other study medication or testing medical devices 30 days before first dose of IXIARO® and during the study period (visit 1 until visit 4)
- Immunodeficiency, organ transplantation, immunosuppressive therapy
- Infection with HIV, hepatitis B or C virus
- Immunosuppressive medication such as corticosteroids ≥0.05 mg/kg/day (>14 d) or immunomodulating drugs (30 d) before the first dose of IXIARO® and during the study period (visit 1 until visit 4) – except inhalation or topical treatment with corticosteroids
- Regular injection with corticosteroids (e.g. intraarticular) within 30 before first dose of IXIARO® and during the study period (visit 1 until visit 4)
- Autoimmune diseases including diabetes type 1. Vitiligo or diseases of the thyroid gland that require thyroid hormone replacement therapy are not excluded.
- Skin cancer within the last 6 months. If the therapy for skin cancer was successfully terminated more than 6 months and the disease is considered to be cured study inclusion is possible. However, these participants must not be vaccinated at the site, where the skin cancer occured previously.
- Every other cancer within the last 5 years. If a cancer therapy was successfully terminated more than 5 years ago and the disease is considered to be cured study inclusion is possible.
- Clinically significant haematological, kidney, liver, lung, CNS, heart or gastrointestinal disease that, according to the principal investigator, was not adequately medically treated within 12 weeks before the first dose of IXIARO® (visit 1).
- Clinically significant menatl illness that cannot be adequately medically controlled.
- Guillan-Barré syndrome (GBS) in the patient history

- Patient history of severe hypersensitivity reactions, particularly against components of the vaccine IXIARO® (e.g. protamine sulfate), anaphylaxis or severe cases of atopy that required emergency treatment or led to hospitalisation previously.
- Urticaria following incesct stings of hymenoptera, medication, physical or other provocation as well as idiopathic urticaria.
- Drug abuse within the last 6 months before first dose of IXIARO® and during the study period (visit 1 until visit 4) including alcohol abuse (>60g per day).
- Individuals unable or not willing to drink less than 60g alcohol within 48h after study medication (i.e. vaccination at visit 1 and 3).
- Other conditions that according to the principal investigator interfere with the study or might incapacitate the individual to complete the study.

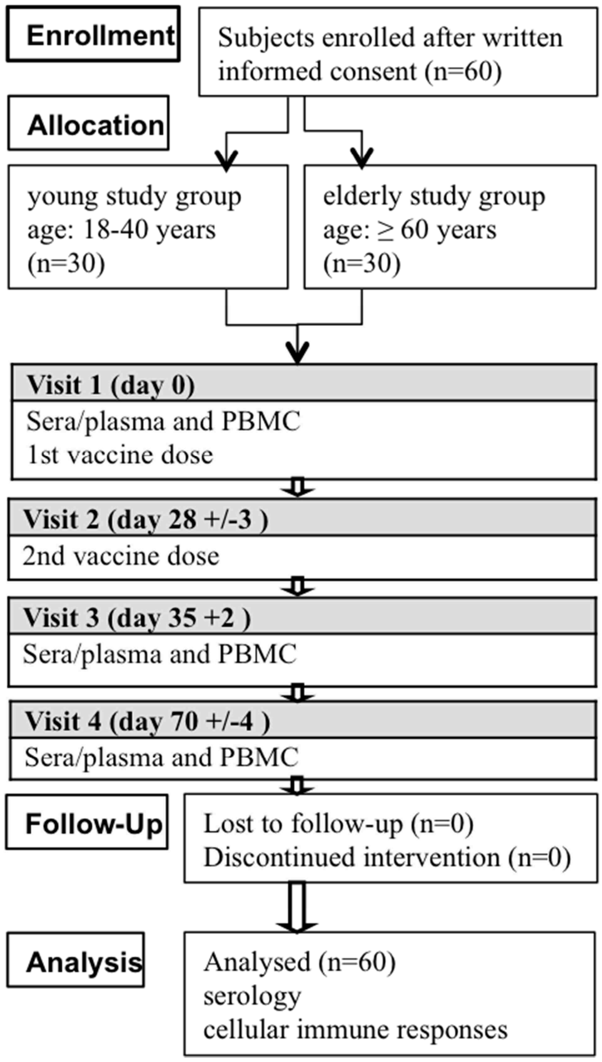

Supplementary Figure 1. Flow chart of study visits and respective procedures.

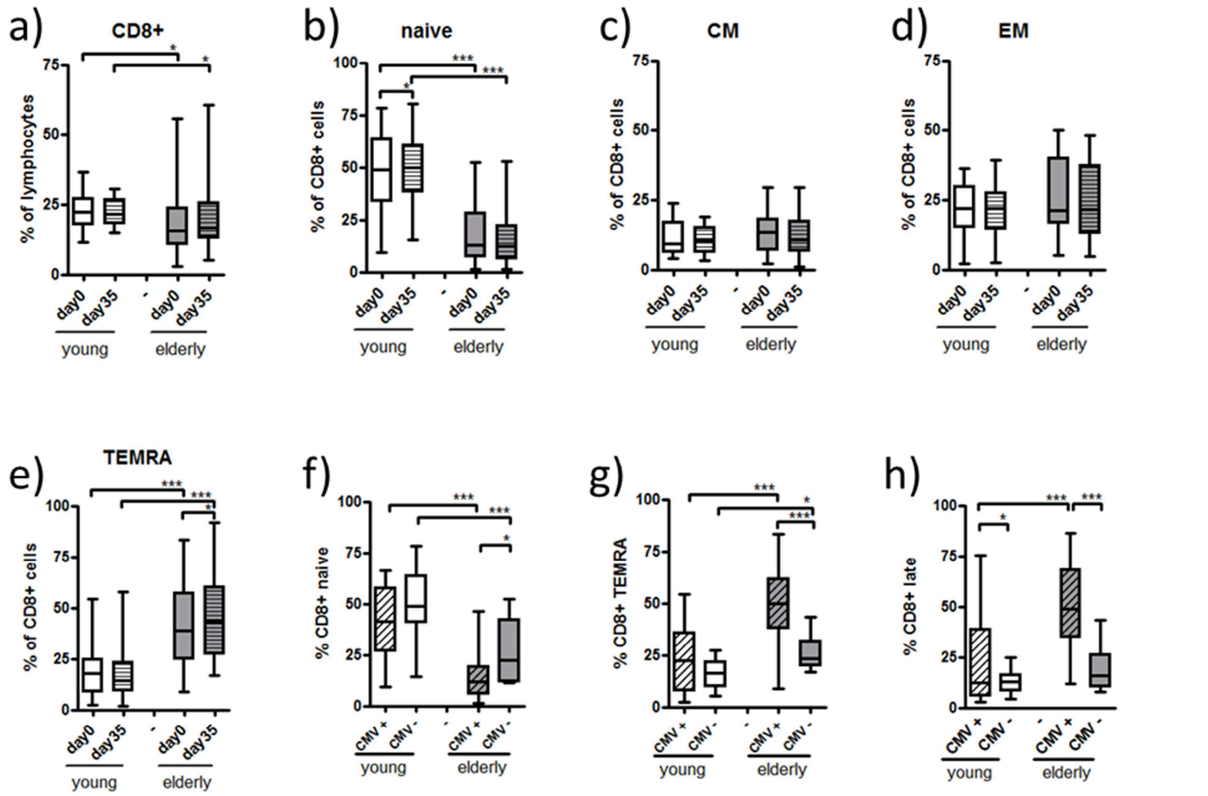

Supplementary Figure 2. **Naive and memory CD8+ cell subsets and correlation with CMV serology.** **(a)** CD8+ T cells were identified as CD4-CD8+ lymphocytes within the live lymphocyte population in a SSC/FSC blot of surface stained PBMC obtained on days 0 and 35. **(b)** Concomitant staining with CD45RA and CCR7 allowed differentiation of naive CD8+ T cells (CD45RA+ CCR7+), **(c)** central memory (CM) CD8+ T cells (CD45RA- CCR7+), **(d)** effector memory (EM) CD8+ T cells (CD45RA- CCR7-) and **(e)** CD8+ T effector memory cells that re-express CD45RA (TEMRA) (CD45RA+ CCR7-) within the CD8+ T cells. Distribution of **(f)** CD8+ naive, **(g)** TEMRA and **(h)** late-differentiated effector cells within CMV-seropositive and -seronegative participants were analysed in samples obtained on day 0 (for gating strategy see Figures 3 and 4). Statistical analysis by General Linear Model with arcsine-transformed percentages. * $p < 0.05$; ** $p < 0.01$; *** $p < 0.001$.

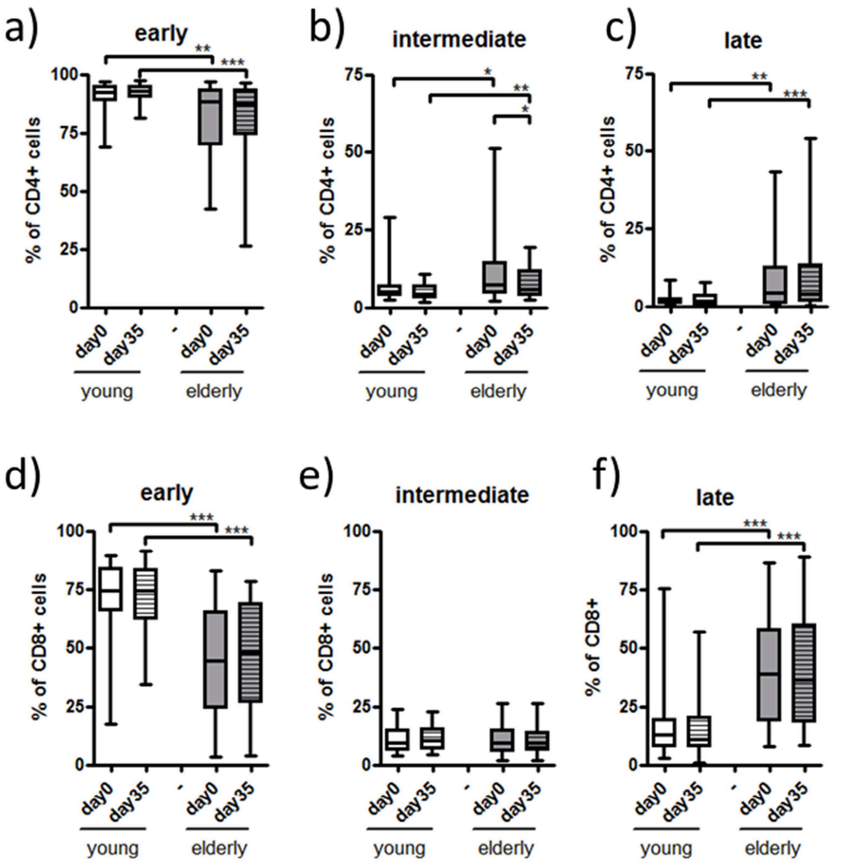

Supplementary Figure 3. **Effector T cell subsets.** **(A)** Early (CD27+ CD28+) **(B)** intermediate (CD27- CD28+) and **(C)** late (CD27- CD28-) differentiated CD4+ T cells were determined within CD4+CD8- gated live lymphocyte population. The CD8+ T cell subsets were evaluated as **(D)** early (CD27+ CD28+), **(E)** intermediate (CD27+ CD28-) and **(F)** late (CD27- CD28-) differentiated within the gated CD4-CD8+ live lymphocyte population. All analyses were performed on PBMC from blood samples drawn on day 0 and 35. Statistical analysis by General Linear Model with arcsine-transformed percentages. * $p < 0.05$; ** $p < 0.01$; *** $p < 0.001$.

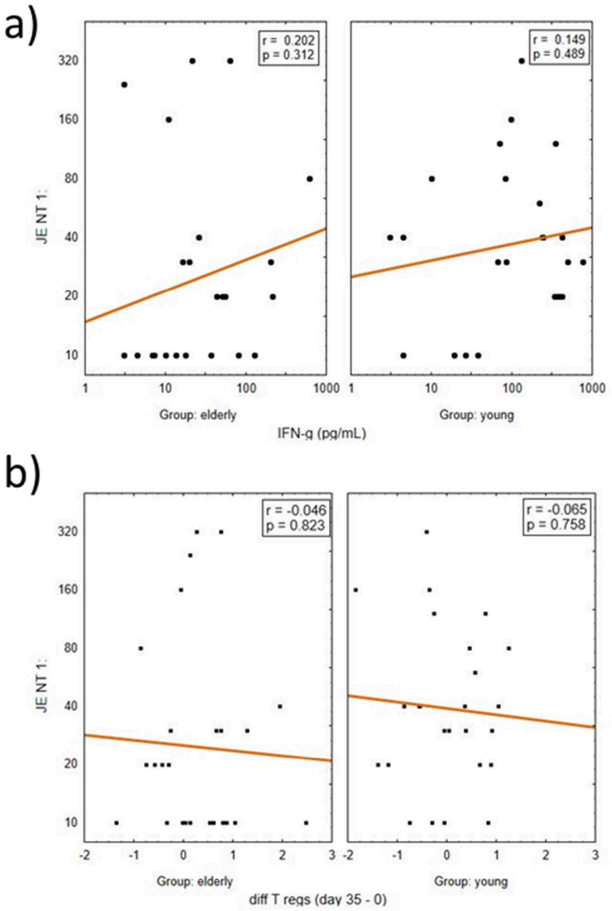

Supplementary Figure 4. Correlation of antibody titres with cellular responses. (a) JE-specific IFN-g production after vaccination (day 35) plotted against JE-specific neutralising antibody titres (day 35) **(b)** change/difference in T regulatory cells between day 0 and day 35 plotted against JE-specific neutralising antibody titres (day 35). Pearson correlation coefficient and associated p-values.
